# Pulmonary Abscess Caused by Neisseria meningitidis: A Case Report

**DOI:** 10.7759/cureus.103535

**Published:** 2026-02-13

**Authors:** Joana Diogo, João Felgueiras, Nuno Mascarenhas, Vasco Firmino, Maria José Simões

**Affiliations:** 1 Pulmonology Department, Unidade Local de Saúde do Arco Ribeirinho, Barreiro, PRT

**Keywords:** alcohol abuse, cavitary lung lesion, meningococcal pneumonia, neisseria meningitidis, smoking

## Abstract

*Neisseria meningitidis* is a Gram-negative diplococcus commonly associated with meningitis or septicemia. However, it may rarely cause pulmonary abscesses, particularly in adults with risk factors such as smoking, chronic alcoholism, or underlying lung disease. The differential diagnosis should include other bacterial etiologies, and laboratory confirmation is essential. Recognition of atypical pulmonary presentations is crucial for appropriate management and mandatory reporting, given the potential for severe progression and the need for chemoprophylaxis among contacts. We report the case of a 60-year-old male, active smoker and chronic alcohol consumer, with a previous history of upper gastrointestinal bleeding requiring antrectomy. The patient presented with six months of anorexia and asthenia, melena of 15 days' duration, and worsening chronic cough. Laboratory findings revealed macrocytic hypochromic anemia, mild hyponatremia, and elevated inflammatory markers. Thoracic CT showed a thick-walled, cavitated mass in the middle lobe suggestive of malignancy. During hospitalization, the patient developed a fever. Bronchoscopy showed purulent, foul-smelling secretions at the middle lobe bronchial emergence. Microbiological analysis of bronchial secretions identified *N. meningitidis*. Directed therapy with intravenous ciprofloxacin (400 mg every eight hours) was initiated, and the patient was maintained under contact isolation, with subsequent clinical and laboratory improvement. Lung abscesses caused by *N. meningitidis* are a rare clinical entity, with few cases described in the literature; meningeal or septicemic involvement is far more common. This case underscores the need to consider*N. meningitidis* in the differential diagnosis of cavitary pulmonary lesions, particularly in patients with risk factors such as chronic smoking and alcoholism. The clinical presentation may mimic lung cancer or abscess, and identification requires microbiological confirmation from sputum or bronchoalveolar lavage samples. Prompt recognition is essential for adequate antibiotic therapy and public health measures. This case highlights the importance of considering *N. meningitidis* in the differential diagnosis of cavitary lung lesions, reinforcing the need for microbiological surveillance, multidisciplinary management, and targeted therapy.

## Introduction

*Neisseria meningitidis *is a strictly human pathogen responsible for invasive meningococcal disease, primarily manifesting as meningitis and septicemia. The main distinction is that meningococcal pneumonia is an acute infection, without cavitation, caused by *N. meningitidis*, whereas a lung abscess is a cavitary lesion with a subacute or chronic course, usually caused by anaerobes or other pathogens. While meningococcal pneumonia occurs in 5-15% of invasive meningococcal disease cases, representing the second most common end-organ manifestation after meningitis, lung abscess formation has been described only in isolated case reports worldwide [1]. A comprehensive review of meningococcal pneumonia identified only 344 cases reported globally between 1906 and 2015, and among these, cavitary lesions or frank abscesses are extraordinarily rare. To our knowledge, only one prior case of meningococcal lung abscess has been documented in recent literature, involving a serogroup X strain with evidence of capsular switching [2].

The pathogenesis of meningococcal pulmonary infection likely differs from typical invasive meningococcal disease. While *N. meningitidis* typically colonizes the nasopharynx and gains access to the bloodstream through mucosal penetration, direct respiratory tract invasion or aspiration may play a more prominent role in pulmonary abscess formation. Humans are the only natural reservoir of N. meningitidis, and the nasopharynx is the site from which meningococci are transmitted by aerosol or secretions to others. Approximately 5-10% of adults are asymptomatic nasopharyngeal carriers of strains of N. meningitidis, most of which are not pathogenic [3].

Several risk factors have been identified for invasive meningococcal disease in adults. Active smoking causes chronic respiratory epithelial damage, while chronic alcohol consumption impairs both local and systemic immune defenses [4-5]. These factors impair mucociliary clearance and local immunity, potentially facilitating lower respiratory tract invasion. Additionally, comorbidities including chronic lung disease and prior complicated surgical procedures may contribute to altered immune function and increased susceptibility to invasive bacterial infections [6-7].

Recognition of meningococcal pulmonary infection carries significant clinical and public health implications. First, the organism's contagious potential requires urgent implementation of droplet precautions to prevent nosocomial transmission. Second, unusual presentations can delay diagnosis and appropriate therapy, potentially leading to adverse outcomes. Third, close contacts of patients with meningococcal disease require antimicrobial chemoprophylaxis to prevent secondary cases.

Here, we present a rare case of a middle-lobe pulmonary abscess due to *N. meningitidis,* diagnosed through bronchoscopy, and review the relevant literature regarding this uncommon manifestation of invasive meningococcal disease.

## Case presentation

A 60-year-old male, independent in activities of daily living, presented to the emergency department with a six-month history of anorexia and asthenia and a two-week history of melena (three episodes per day) and worsening chronic cough. He denied chest pain, hemoptysis, or documented weight loss.

Past medical history included massive upper gastrointestinal bleeding in 2019 secondary to a gastroduodenal artery rupture, surgically treated by antrectomy and duodenorrhaphy. The postoperative period was complicated by hemorrhagic shock, pulmonary embolism, nosocomial pneumonia, and acute respiratory distress syndrome requiring invasive mechanical ventilation. He completed seven months of oral anticoagulation, discontinued in 2020.

He reported daily smoking since age 16 (approximately one pack per day, 44 pack-years) and regular alcohol intake of approximately one liter of red wine plus spirits per day. He was not on chronic medication and had no known drug allergies. There was no history of recent dental procedures or known immunodeficiency.

On admission, the patient was alert and hemodynamically stable with the following vital signs: blood pressure 128/76 mmHg, heart rate 88 beats per minute, respiratory rate 18 breaths per minute, temperature 36.8°C, and oxygen saturation 96% on room air. Physical examination revealed pallor but no signs of active gastrointestinal bleeding. Chest examination showed decreased breath sounds and scattered coarse crackles over the right mid-lung field. Cardiac examination was unremarkable. The abdomen was soft, non-tender, with healed surgical scars and no organomegaly. There was no peripheral lymphadenopathy or skin lesions.

Laboratory investigations on admission revealed the following abnormalities: hemoglobin 9.2 g/dL (reference range 13.5-17.5 g/dL) and mean corpuscular volume 102 fL (reference range 80-100 fL), consistent with macrocytic anemia. Serum sodium was 130 mmol/L (reference range 135-145 mmol/L). C-reactive protein was markedly elevated at 28.1 mg/dL (reference range 0.5 mg/dL). White blood cell count, platelet count, liver function tests, and renal function tests were within normal limits.

**Figure 1:**
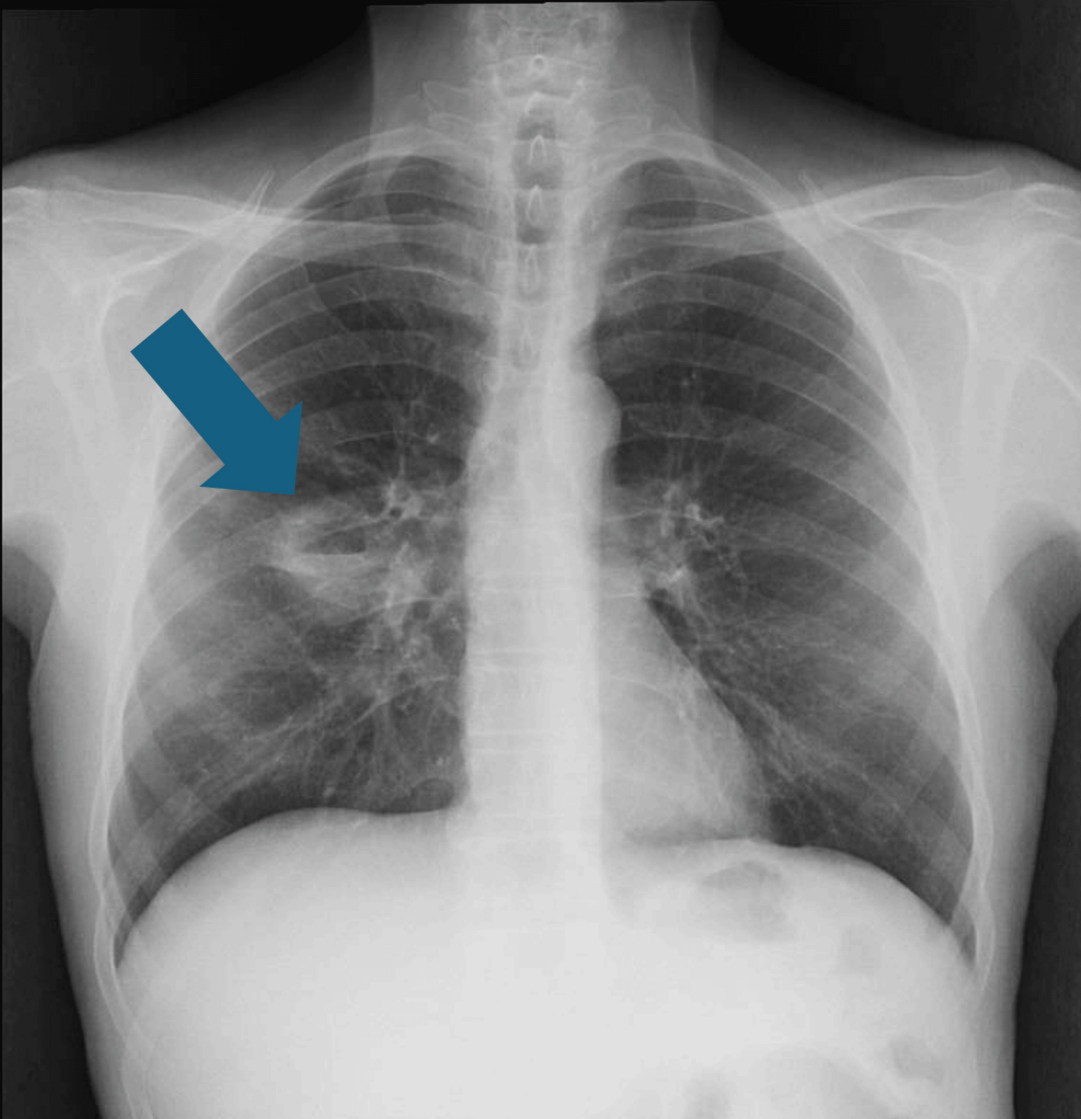
Posteroanterior chest radiograph demonstrating a well-defined cavitary lesion in the right middle lobe, characterized by a radiolucent area with a thick, irregular wall and possible internal air–fluid level. The blue arrow indicates the cavitary lesion.

**Figure 2:**
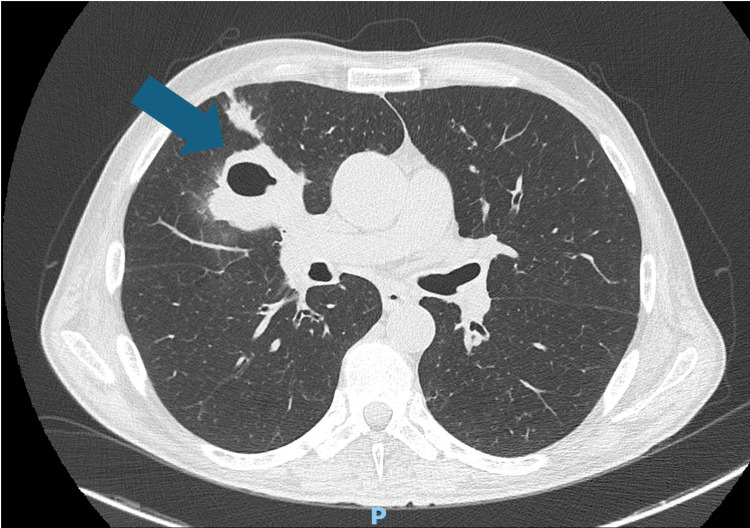
Chest CT scan showing a thick-walled cavitary lesion measuring 5.2 × 4.5 cm in the right middle lobe, with irregular internal margins. The surrounding parenchyma shows adjacent consolidation suggestive of inflammatory changes. The blue arrow indicates the cavitary lesion.

Posteroanterior chest radiograph showed evidence of a cavitary lesion in the middle lobe (Figure 1), and chest computed tomography (CT) scan (Figure 2) demonstrated a thick-walled cavitary lesion in the right middle lobe, with irregular internal margins. The radiographic appearance was initially interpreted as suggestive of a possible neoplastic lesion, given the patient's significant smoking history and the morphology of the mass. No mediastinal lymphadenopathy or pleural effusion was identified.

Sputum samples were collected and were negative for *Mycobacterium tuberculosis* by acid-fast bacilli smear and culture. Sputum cytology was non-diagnostic.

Given the cavitary lesion and persistent systemic symptoms, the patient was admitted for etiological investigation and concurrent evaluation of melena. Upper gastrointestinal endoscopy revealed Los Angeles grade C esophagitis without active bleeding; proton-pump inhibitor therapy (esomeprazole 40 mg) twice daily was initiated.

During hospitalization on day 3, the patient developed fever up to 39°C. Empirical broad-spectrum antibiotic therapy with piperacillin-tazobactam was initiated for suspected healthcare-associated pneumonia. Bronchofibroscopy was performed on day 4 to obtain diagnostic samples from the cavitary lesion. The procedure revealed abundant purulent, foul-smelling secretions at the entrance of the middle lobe bronchus and inflamed, friable mucosa. Bronchial aspirates were collected for cytological, microbiological, and histopathological evaluation.

Cytological examination was negative for malignancy. Microbiological culture of bronchial secretions yielded *N. meningitidis* after 48 hours of incubation; blood cultures obtained prior to antibiotic initiation remained sterile after five days of incubation. Upon microbiologic confirmation of meningococcal infection, therapy was switched to intravenous ciprofloxacin 400 mg every eight hours, according to antimicrobial susceptibility results. The isolate demonstrated susceptibility to ciprofloxacin, ceftriaxone, and penicillin. Serogroup determination was not performed at the treating institution.

The patient was immediately placed under contact and droplet isolation precautions. Healthcare workers who had contact with the patient prior to isolation were identified and offered antimicrobial chemoprophylaxis in coordination with the hospital's infection control team. Close household contacts were also identified and provided with appropriate chemoprophylaxis.

The patient showed progressive clinical improvement following targeted antimicrobial therapy. Fever subsided within 72 hours of ciprofloxacin initiation, and inflammatory markers demonstrated a downward trend. C-reactive protein decreased to 8.4 mg/dL by day 7 and 2.1 mg/dL by day 14. Cough gradually resolved, and the patient's constitutional symptoms improved. Follow-up chest imaging on day 10 showed partial regression of the cavitary lesion with decreased surrounding inflammatory changes.

The patient was discharged on day 16 in clinically stable condition to complete an additional four-week course of oral ciprofloxacin 750 mg twice daily under outpatient follow-up, for a total treatment duration of six weeks.

At outpatient follow-up four weeks post-discharge, the patient was asymptomatic with complete resolution of cough and constitutional symptoms. Physical examination was unremarkable. Laboratory investigations demonstrated normalization of inflammatory parameters with a C-reactive protein of 0.5 mg/dL. Hemoglobin had improved to 11.8 g/dL with ongoing nutritional support and alcohol abstinence counseling.

Repeat chest CT demonstrated near-complete resolution of the cavitary lesion, with only minimal residual scarring in the right middle lobe and no new pulmonary findings. The patient was advised to continue smoking cessation efforts and alcohol abstinence, and to maintain regular follow-up with his primary care physician.

## Discussion

This case represents an exceptionally rare manifestation of invasive meningococcal disease presenting as a cavitary pulmonary abscess [8]. While *N. meningitidis* is well-recognized as a leading cause of bacterial meningitis and meningococcemia, respiratory involvement beyond pneumonia is extraordinarily uncommon [9-10]. To our knowledge, only one prior case of meningococcal lung abscess has been documented in recent literature, involving a serogroup X strain with evidence of capsular switching from serogroup B [11-12].

Pathogenesis and risk factors

The pathogenesis of meningococcal pulmonary abscess likely differs from typical invasive meningococcal disease. While *N. meningitidis *typically colonizes the nasopharynx and gains access to the bloodstream through mucosal penetration, direct respiratory tract invasion or aspiration may play a more prominent role in pulmonary abscess formation. Our patient presented multiple risk factors that likely contributed to this atypical presentation [11-12]. Active smoking and chronic alcohol consumption are established risk factors for invasive meningococcal disease. A recent systematic review and meta-analysis identified smoking and alcohol abuse as significant risk factors for contracting invasive meningococcal disease. Additionally, our patient's history of complicated upper gastrointestinal surgery with prolonged postoperative course, including acute respiratory distress syndrome requiring mechanical ventilation, may have resulted in chronic aspiration risk, altered local pulmonary immunity, or structural lung damage predisposing to localized infection [11-12].

Clinical presentation and diagnostic challenges

The clinical presentation initially suggested malignancy, given the patient's smoking history, chronic constitutional symptoms, and radiographic appearance of a thick-walled cavitary mass. This diagnostic challenge underscores an important clinical lesson: atypical pathogens, including *N. meningitidis*, must be considered in the differential diagnosis of cavitary lung lesions, particularly in patients with risk factors for invasive bacterial infections [13-14]. The differential diagnosis of cavitary lesions in smokers with alcohol use disorder is broad and includes primary lung cancer, tuberculosis, anaerobic bacterial abscesses (often polymicrobial), fungal infections (including *Aspergillus* and endemic mycoses), septic emboli, and vasculitic processes. The presence of purulent, foul-smelling secretions at bronchoscopy suggested bacterial abscess, but the isolation of *N. meningitidis* was unexpected and highlights the importance of comprehensive microbiological evaluation [13-14]. Meningococcal pneumonia typically occurs in 5-15% of invasive meningococcal disease cases. However, the organism is rarely isolated from respiratory specimens in the absence of bacteremia. In reported cases of meningococcal pneumonia, blood cultures are often positive even when sputum cultures are negative. The successful isolation of *N. meningitidis* from bronchial secretions in our patient, in the absence of positive blood cultures, represents an unusual microbiological finding that may reflect the localized nature of the infection within the abscess cavity rather than systemic dissemination [13-14].

Microbiological considerations

Serogroup determination and molecular characterization of the isolate would have been valuable, as certain serogroups and clonal complexes demonstrate distinct epidemiological patterns and clinical presentations. Serogroup Y has been associated with pneumonia and accounted for 44.2% of identified isolates in a review of meningococcal pneumonia cases. Serogroup W-135 has also been associated with pneumonia and arthritis. The single documented case of meningococcal lung abscess involved a serogroup X strain with evidence of capsular switching from serogroup B, suggesting that genetic recombination events may contribute to unusual tissue tropism [13-14].

The absence of bacteremia in our patient, despite the presence of a large pulmonary abscess, is noteworthy. This finding suggests that the infection remained localized to the respiratory tract without systemic dissemination, which may have contributed to the relatively favorable clinical course. The mechanisms by which *N. meningitidis* establishes infection within the lung parenchyma without causing bacteremia remain speculative but may involve direct aspiration of colonized nasopharyngeal secretions into a structurally or immunologically compromised area of lung tissue [13-14].

Treatment and infection control

The initiation of targeted ciprofloxacin therapy (400 mg IV three times daily) was appropriate based on culture results and susceptibility testing. While third-generation cephalosporins are typically recommended as empiric therapy for suspected meningococcal disease, fluoroquinolones demonstrate excellent activity against *N. meningitidis *and achieve good tissue penetration [13-14]. However, emerging ciprofloxacin resistance has been documented in some regions, emphasizing the importance of antimicrobial susceptibility testing to guide definitive therapy [15-16]. The optimal duration of treatment for meningococcal pulmonary abscess is not established, though uncomplicated meningococcal pneumonia can be treated effectively with five to seven days of appropriate antibiotics [17-18].

Critical infection control measures were appropriately implemented in this case. The patient was placed under contact and droplet precautions, which are essential for preventing nosocomial transmission of *N. meningitidis*. Healthcare workers should maintain droplet precautions (wearing masks) when caring for patients with suspected or confirmed meningococcal respiratory infections until 24 hours after initiation of effective antimicrobial therapy. Close contacts of the patient should receive chemoprophylaxis with rifampin, ciprofloxacin, ceftriaxone, or azithromycin, ideally within 24 hours of case identification. The definition of close contacts includes household members and individuals with prolonged proximity (within 3 feet for ≥8 hours) or direct exposure to respiratory secretions [17-18].

Limitations and future directions

This case report has limitations inherent to single-case observations. Serogroup determination and molecular characterization of the isolate were not reported, which would have provided valuable epidemiological and pathogenic insights. In addition, the mechanism by which *N. meningitidis* establishes infection within the lung parenchyma remains speculative. Future research should focus on identifying virulence factors that enable meningococcal strains to cause localized pulmonary infections and determining whether specific genetic lineages demonstrate enhanced tropism for respiratory tissues [19-20]. In conclusion, this case expands the recognized clinical spectrum of invasive meningococcal disease to include pulmonary abscess formation. Clinicians should maintain awareness of this rare but serious manifestation, particularly in patients with predisposing risk factors. Prompt microbiological diagnosis, targeted antimicrobial therapy, and rigorous infection control measures are essential for optimal patient outcomes and prevention of secondary transmission [19-20].

## Conclusions

This case describes a rare presentation of invasive meningococcal disease as a cavitary pulmonary abscess in an adult with multiple predisposing risk factors, expanding the recognized clinical spectrum of Neisseria meningitidis infection. Smoking, chronic alcohol use, and prior surgical history likely contributed to impaired pulmonary defenses, facilitating localized infection. The initial radiological appearance mimicking lung malignancy highlights the limitations of imaging alone and underscores the importance of comprehensive microbiological evaluation in patients with cavitary lung lesions. Isolation of N. meningitidis from bronchial secretions without bacteremia suggests localized disease, which may have contributed to the favorable clinical outcome following targeted antimicrobial therapy. Limitations include the absence of serogroup and molecular characterization and an incomplete understanding of the mechanisms underlying pulmonary tropism. In conclusion, meningococcal pulmonary abscess is an exceptionally rare but clinically significant manifestation. Clinicians should consider this diagnosis in patients with relevant risk factors and atypical lung lesions. Early recognition, thorough diagnostic investigation, and prompt antimicrobial treatment are essential to optimize outcomes and prevent secondary transmission.
